# Lactate-Modulated Induction of THBS-1 Activates Transforming Growth Factor (TGF)-beta2 and Migration of Glioma Cells *In Vitro*

**DOI:** 10.1371/journal.pone.0078935

**Published:** 2013-11-01

**Authors:** Corinna Seliger, Petra Leukel, Sylvia Moeckel, Birgit Jachnik, Claudio Lottaz, Marina Kreutz, Alexander Brawanski, Martin Proescholdt, Ulrich Bogdahn, Anja-Katrin Bosserhoff, Arabel Vollmann-Zwerenz, Peter Hau

**Affiliations:** 1 Department of Neurology and Wilhelm Sander-NeuroOncology Unit, University Hospital Regensburg, Regensburg, Germany; 2 Institute of Pathology, University of Regensburg, Regensburg, Germany; 3 Department of Hematology and Oncology, University of Regensburg, Regensburg, Germany; 4 Department of Neurosurgery, University of Regensburg, Medical School, Regensburg, Germany; 5 Institute for Functional Genomics, Biopark I, Regensburg, Germany; University Hospital of Heidelberg, Germany

## Abstract

**Background:**

An important phenomenon observed in glioma metabolism is increased aerobic glycolysis in tumor cells, which is generally referred to as the Warburg effect. Transforming growth factor (TGF)-beta2, which we previously showed to be induced by lactic acid, is a key pathophysiological factor in glioblastoma, leading to increased invasion and severe local immunosuppression after proteolytic cleavage from its latency associated peptide. In this study we tested the hypothesis, that lactate regulates TGF-beta2 expression and glioma cell migration via induction of Thrombospondin-1 (THBS-1), a TGF-beta activating protein.

**Methods:**

Lactate levels were reduced by knockdown of LDH-A using specific small interfering RNA (siRNA) and competitive inhibition of LDH-A by sodium oxamate. Knockdown of THBS-1 was performed using specific siRNA. Western Blot, qRT-PCR, and ELISA were used to investigate expression levels of LDH-A, LDH-B, TGF-beta2 and THBS-1. Migration of cells was examined by Spheroid, Scratch and Boyden Chamber assays.

**Results:**

Knockdown of LDH-A with subsequent decrease of lactate concentration leads to reduced levels of THBS-1 and TGF-beta2 in glioma cells. Lactate addition increases THBS-1 protein, leading to increased activation of TGF-beta2. Inhibition of THBS-1 reduces TGF-beta2 protein and migration of glioma cells. Addition of synthetic THBS-1 can rescue reduced TGF-beta2 protein levels and glioma cell migration in siLDH-A treated cells.

**Conclusion:**

We define a regulatory cascade between lactate, THBS-1 and TGF-beta2, leading to enhanced migration of glioma cells. Our results demonstrate a specific interaction between tumor metabolism and migration and provide a better understanding of the mechanisms underlying glioma cell invasion.

## Introduction

More than 60 years ago, Otto Warburg described the phenomenon of “aerobic glycolysis” [1,2]. In solid tumors, despite sufficient oxygen, lactate dehydrogenase (LDH) metabolizes pyruvate to lactate. A subtype of LDH, LDH-A, is up-regulated in glioma cells [3] which leads to production of the enzymes LDH IV and V, that mainly metabolize pyruvate to lactate, whereas higher expression of LDH-B leads to formation of LDH I, II and III catalysing the oxidation of lactate to pyruvate. Human *in vivo* ^18^fluor-deoxy-glucose positron emission tomography (FDG-PET) and proton magnetic resonance spectroscopy (^1^H-MRS) reveal elevated glucose [4] and lactate [5] uptake in gliomas, related to their grade. These observations indicate increased glycolysis. As a consequence, the end product of glycolysis, lactate, is produced and secreted in co-transport with protons by monocarboxylate transporters (MCTs). This leads to accumulation of lactate in the tumor microenvironment and to acidification [6]. Consecutive decreases in the extracellular pH [7] lead to several effects including increased migration of tumor cells [8,9]. Based on these results, glioblastoma is a paradigmatic tumor for the investigation of the role of glycolysis in tumor cell invasion.

TGF-beta2 is a key factor in glioblastoma pathogenesis [10]. Expression of TGF-beta2 correlates with glioma grade [11]. In tumor tissue TGF-beta2 leads to immune suppression as well as increased tumor growth, invasion, and metastasis [12,13]. Besides, TGF-beta2 serves as a potent inducer of angiogenesis [14] and of extracellular matrix-molecules [15].

Lactic acid has been shown to promote glioma migration by TGF-beta2 dependent regulation of matrix metalloproteinase-2 (MMP-2) [16]. However, the mechanism how lactic acid influences expression of TGF-beta2 and the role of activators of TGF-beta, such as THBS-1, has not been investigated in this context so far. 

THBS-1 is an extracellular matrix molecule involved in glioma migration, invasion, suppression of angiogenesis and activation of TGF-beta [17,18]. THBS-1 belongs to a group of structurally related glycoproteins, named THBS-1, THBS-2, THBS-3, THBS-4 und THBS-5 / cartilage oligomeric matrix protein [19]. Physiologically, THBS-1 is secreted by platelets; however, a number of other cells including smooth muscle cells, astrocytes, endothelial cells and various tumor cells are also able to produce THBS-1 [20]. The structural complexity of THBS-1 that consists of an amino-terminal heparin-binding domain, a procollagen-like domain, 3 type I (properdin-like) repeats, 3 type II (epidermal growth factor-like) repeats, 7 type III (calcium-binding) repeats and a carboxy-terminal cell binding domain explains the various functions of this molecule predominantly mediated by different cell and extracellular matrix binding sites [17,21]. Kawataki et al. were able to provide evidence that THBS-1 and TGF-beta2 correlate with glioma grade, showing higher expression levels in malignant high-grade gliomas, than in low-grade gliomas [22]. Further, THBS-1 increases glioma migration through TGF-beta2 dependent and independent pathways [23]. Activation of TGF-beta2 is induced by binding of THBS-1 to TGF-beta´s latency associated peptide, permitting proteolytic cleavage [24,25].

We showed earlier that incubation of glioblastoma cell lines with lactic acid induces the expression of TGF-beta2 [16]. Furthermore previous experiments indicate that down-regulation of LDH-A by specific siRNA (siLDH-A), followed by decreased lactate levels, suppresses TGF-beta2. By means of microarray analysis (Affymetrix U133; Affymetrix, Santa Clara, CA, USA; unpublished results), we could also demonstrate that decreased lactate down-regulates THBS-1 mRNA, a pivotal TGF-beta activating protein. We therefore hypothesized that LDH-A-mediated changes in local lactate levels modulate the activation of TGF-beta2, which is mediated, at least in part, via THBS-1. 

## Materials and Methods

### Culture of glioma cell lines

Glioma cell lines and primary cultures were used for *in vitro* experiments. The human glioblastoma cell lines U87MG and A172 were obtained from American Type Culture Collection (Manassas, VA, USA). The gliomas named as “HTZ” were primary tumor cell cultures derived from surgical specimens of human glioblastomas, as described [15]. Tumor cells were maintained as monolayer cultures in Dulbecco’s Modified Eagle Medium (DMEM; PAA, Pasching, Austria), supplemented with 5% fetal calf serum (FCS; PAA, Pasching, Austria) at 37°C, 5% CO_2_, 95% humidity in a standard tissue culture incubator. 

Core results were verified in cultures of brain tumor initiating cells (BTIC, supplementary data) [26,27], that were established from resections of untreated human malignant gliomas. Progenitor features of BTIC lines were verified by multiparametric flow cytometry [27] using established markers of BTIC (CD133, CD15, CD44, A2B5), markers of progenitor cells (Nestin, Sox2, GFAP), by clonogenicity assays and partly by tumor take assays in the nude mouse model. RAV20 cells (anaplastic astrocytoma, IDH1_mut_, MGMT promoter methylation not performed, CD133+, CD15+, CD44 n.d., A2B2-, Nestin_high_, Sox2_moderate_, GFAP_low_, proneural phenotype, clonogenicity +, tumor take +) were derived from a 30 year-old patient, RAV21 cells (primary glioblastoma, IDH1_wt_, MGMT promoter methylation 100%, CD133-, CD15-, CD44+, A2B2+, Nestin_low_, Sox2_low_, GFAP_moderate_, mesenchymal phenotype, clonogenicity +, tumor take +) were extracted from a 46-year old patient. BTIC cell lines with moderate to high proliferation rates, expression of THBS-1 and TGF-beta2, and a functional TGF-beta signaling pathway [28] verified by Smad2 phosphorylation after TGF-beta treatment, were used for this project. Use of human material has been approved by the institutional review board of the University of Regensburg, Germany (No° 11-103-0182). All participants provided written informed consent to participate in this study. Human dermal fibroblasts were provided by Dr. Tim Maisch, Department of Dermatology, University Hospital Regensburg. 

### Treatment of glioma cell lines with lactate, lactic acid and sodium oxamate

Glioma cell lines and controls were treated with sodium lactate (Sigma, Germany), lactic acid (Sigma, Germany), HCl as pH control (Merck, Germany) and sodium oxamate (Fluka, Germany). Cells were seeded in 6-well plates, maintained in serum-free conditions over night and incubated with different concentrations (0, 5, 10, 20 mM) of lactate for 24 hours. After 2 hours and before harvest, pH was controlled and if necessary adjusted to pH 7.1. Starved cells were treated by different concentrations of sodium oxamate (Fluka, Germany) for 24 h. Cells and supernatants were harvested to prepare total RNA or protein as described below. Cell lysates and supernatants of untreated cells were used as controls in all assays.

### Magnetic resonance spectroscopy

Metabolic analysis of lactate and glucose before and after treatment was performed by nuclear magnetic resonance spectroscopy (^1^H-NMR, Bruker Avance 600 MHz spectrometer, Bruker, Germany) as published [29]. Briefly, 600 µl of cell culture supernatants containing 50 µM dimethyl-silapentanesulfonate (DSS, internal standard for quantification) and 10% D_2_O were filled into standard 500 µl NMR tubes (Norell Inc., Landisville, NJ). One-dimensional NOESY spectra (Bruker pulse sequence *noesygppr1d*) were acquired with a repetition time of 10 seconds, a mixing time of 10 ms, and a weak presaturation pulse of 8.2 seconds to suppress the water signal. Sixteen scans with 32,768 datapoints and a sweepwidth of 8993 Hz were accumulated followed by an exponential line broadening of 0.3 Hz. The spectra were corrected for baseline and phase artifacts manually, and the frequency of the methyl peak of DSS was set to 0.00 ppm. Quantities of lactate and glucose were obtained by fitting model spectra of known metabolites (biorefcode database, Bruker, Germany) to the data by means of a standard Matlab (The MathWorks, Natick, MA, USA) optimization routine.

### Measurement of LDH V activity and extracellular lactate concentration

2x10^5^ glioma cells or fibroblasts were seeded in 6-Well plates and treated with siLDH-A or sodium oxamate for 24 hours. Then, cells were counted and 5x10^3^ of these cells were transferred into 96-Well plates and lysed to measure LDH V activity using the cytotox assay (Promega, Germany) according to the manufacturer’s instructions. Lactate levels in cell culture supernatants were measured with a COBAS analyzer (Roche, Germany), measuring 2x10^3^ of the differentially treated cells.

### Transient transfection of glioma cell lines for LDH-A, LDH-B and THBS-1 knockdown

Transient transfection of glioma cell lines and BTIC using Lipofectamin 2000 (Invitrogen GmbH, Darmstadt, Germany) was performed with 0.1-0.2 µM siRNA against LDH-A (siLDH-A), LDH-B (siLDH B) and THBS-1 (siTHBS-1) to evaluate mRNA level changes after 12, 24, 48, and 72 hours and to generate about 90% knockdowns of the respective proteins.

### pH measurement

For pH measurement, a pH-electrode (HANNA Instruments, Kehl am Rhein, Germany) was calibrated at 37°C, and the pH of the cell supernatant was measured directly in the incubator immediately after opening of the incubator and cell culture flask.

### THBS-1 and TGF-beta2 stimulation assays

To investigate the effect of exogenous THBS-1 and TGF-beta2 on the function of glioma cells we performed *in vitro* stimulation assays. We seeded 2x10^5^ glioma cells in 6-well plates. After 24 hours, triplicates of subconfluent cell layers were treated with 20 ng/ml rhTGF-beta2 protein (R&D Systems GmbH, Germany) or recombinant human THBS-1 protein (R&D Systems GmbH, Germany, Asn 19 - Pro 1170; Accesion #P0799) [30] as indicated in the text and incubated for 72 hours. Cells and supernatants were harvested to prepare total RNA or protein as described below or to investigate the cells in Scratch Migration Assays as indicated below.

### RNA isolation

For RNA isolation, cells were incubated in 6-well plates (2 x10^5^ cells per 2 ml). Total RNA was isolated using the RNeasy Mini Kit (Qiagen, Germany) and reverse transcribed using M-MLV reverse transcriptase (Promega, Germany) following the manufacturer’s instructions.

### Polymerase Chain Reaction (PCR) and Quantitative Real Time PCR (qRT-PCR)

Primers to detect transcripts of interest were: LDH-A (forward: 5'-GGT TGG TGC TGT TGG CAT GG-3’, reverse: 5'-TGC CCC AGC CGT GAT AAT GA-3‘, Genbank Accession No: NM_001135239.1), THBS-1 (forward: 5’-CAG AAG GAC TCT GAC GGC G-3’, reverse: 5’-GAA TCA TCT GGA ATC GGC GG-3’, Genbank Accession No: NM_003246.2), TGF-beta2 (forward: 5' CAC CAT AAA GAC AGG AAC CTG -3'; reverse: 5´- GGA GGT GCC ATC AAT ACC TGC -3´, Genbank Accession No: NM_003238), and RPLPO (large ribosomal protein) (forward: 5’-CTG TCT GCA GAT TGG CTA CCC-3’, reverse: 5’-GAT GGA TCA GCC AAG AAG GC-3’, Genbank Accession No: NM_001002.3) as a positive control in all assays. Annealing temperatures were optimized for each primer pair. PCR products were analyzed on a 1% agarose gel and visualized with ethidium bromide staining. 

Quantification of mRNA expression was performed by real-time PCR (Mx3000P Quantitative PCR [qPCR] System, Stratagene, Germany) based on SYBR-Green I fluorescence (QuantiFast SYBR Green PCR Kit Qiagen, Germany). Briefly, five serial twofold dilutions of cDNA were amplified in triplicate to construct standard curves for both the target gene and the endogenous reference (18s or RPLPO, ribosomal protein, large, P0). For each reaction, melting curves and agarose gel electrophoresis of PCR products were used to verify the identity of the amplification products. The target gene amount was divided by the endogenous reference (18s or RPLPO) amount to obtain a normalized target value. Each of the experimental normalized values was divided by the normalized control (untreated) sample value to generate the relative expression levels in fold changes.

### Protein isolation

To confirm knockdown effects of LDH-A and THBS-1 on the protein level, whole-cell lysates were prepared with RIPA buffer, and the samples (10-30 µg) were subjected to Western blotting on a denaturing 10% SDS-PAGE.

### TGF-beta2 ELISA

For quantitative determination of activated human TGF-beta2 concentrations in cell culture supernatants, the quantitative sandwich enzyme linked immunoassay technique was used with a commercially available human TGF-beta2 specific immunoassay kit (R&D Systems, Minneapolis, MN). The minimum detectable dose of TGF-beta2 was less than 7.0 pg/ml. The assay was performed in triplicate according to the manufacturer’s instructions. 

### Western Blot

For detection of all other proteins, SDS-PAGE and Western Blot on a nitrocellulose membrane (Bio-Rad Laboratories, Munich, Germany) was performed. Monoclonal antibodies specific for LDH-A, THBS-1, TGF-beta2 (Santa Cruz Biotechnology, Germany), βActin (Sigma-Aldrich, Germany), pSmad2 (Calbiochem, Germany) and LDH-B (Abnova, Germany) were used. Immunocomplexes were visualized using horseradish peroxidase-conjugated antibodies (BD Bioscience, Heidelberg, Germany) followed by enhanced chemoluminescence (ECL) with Immobilon Western HRP Substrate (Millipore GmbH, Schwalbach, Germany) and detection on Hyperfilm ECL 1 (Amersham, Munich, Germany).

### Proliferation assays

Proliferation of glioma cells was determined by counting viable cells using a Neubauer chamber. Non-viable cells were identified by trypan blue. Every experiment was performed in triplicate and repeated twice.

### Scratch Migration Assay

In order to determine glioma cell migration with the Scratch migration assay, 2 x 10^5^ glioma cells were seeded in each well of a 6-well plate. An artificial gap, a so called “scratch” was created in the confluent cell monolayer using a pipette tip. Images were captured at the beginning and regular intervals (24, 48, 72 and 96 hours) during cell migration into the scratch, and the area of the “scratch” was measured in µm^2^ and compared between images in order to determine the rate of cell migration as ∆µm^2^. Assays were performed in triplicate, repeated twice and evaluated by a blinded investigator.

### Spheroid Migration Assay

Tumor spheroids were initiated by seeding 3-8 x 10^3^ cells in agar-coated wells. Mature spheroids with a mean diameter of 200-250 µm were explanted to uncoated 96 well plates. Spheroids were allowed to migrate for 1-5 days using the earliest time point where migration was visible to prevent a dilution of the effect by enhanced proliferation of cells. The diameter of the area covered by cells migrating away from a spheroid was photographed and the diameter was measured manually by a blinded investigator using the greatest diameter. Bovine serum albumin (BSA) was used as a control protein. Assays were performed in triplicate and repeated twice.

### Boyden Chamber Migration Assay

A suspension of 200,000 tumor cells per ml (total volume: 200 µl) was pipetted in the upper chamber of the Boyden device (Becton Dickinson, USA). The lower chamber was loaded with 210 µl of a chemo-attractant consisting of cell culture medium that had been harvested after a 24-h incubation of fibroblasts grown in DMEM. The chambers are divided by an uncoated membrane with pores of 8 µm diameter. After 4 h of incubation, the number of cells that had migrated to the lower side of the membrane was counted after staining with hematoxilin and eosin. Five visual fields were counted by a blinded investigator on each filter of a triplet and were evaluated calculating means of migrated cells and standard deviation. 

### Statistics

The Student`s t-test (paired/equal variance) was used to compare the results (mean values and SDs) of control *vs.* treated cell samples. The level of significance was set at *P<0.05, **P<0.01, and ***P<0.001. 

## Results

### Expression of LDH-A and secretion of lactate in glioma cell lines

**Figure 1:**
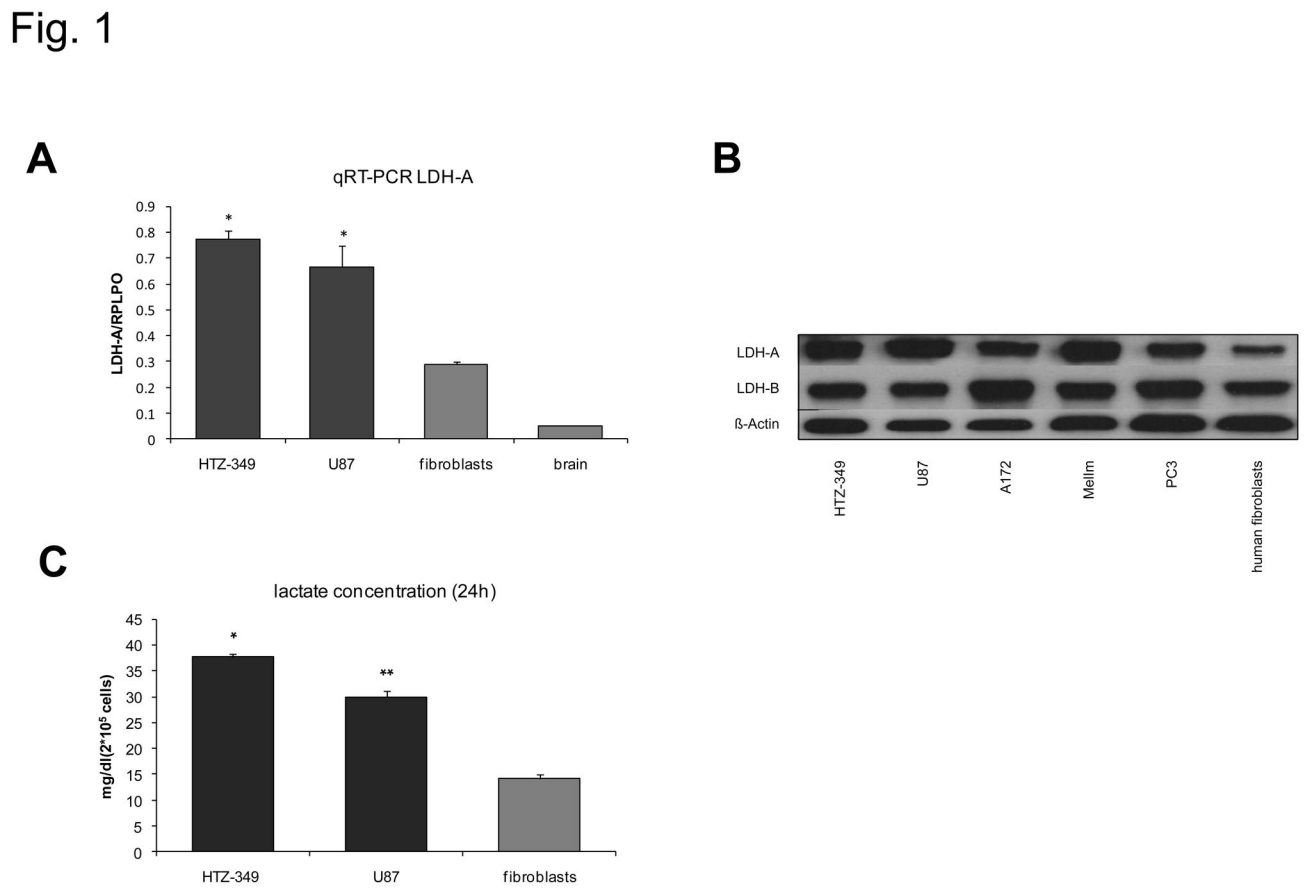
Glioma cell lines express LDH-A and secrete lactate. Glioma cell lines HTZ-349 and U87 were cultured under comparable conditions. Using qRT-PCR, mRNA levels of LDH-A were determined using specific primers for LDH-A (A). Additionally, total protein was isolated and LDH-A and LDH-B protein expression was determined in Western Blot analysis (B). Glioma cell lines have increased mRNA levels of LDH-A in comparison to fibroblasts and human brain RNA (A, p < 0.05*) and show increased protein expression, as do a melanoma cell line (MelIm) and a prostatic cancer cell line (PC3) (B). To examine lactate expression cell culture supernatants (2*10^5^ cells/well) were collected and lactate levels were measured (C). Glioma cell lines secrete higher levels of lactate if compared to fibroblasts (U87, p < 0.01**; HTZ-349 p < 0.05*).

To verify a relevant activity of LDH-A in glioma cell lines, we evaluated baseline LDH-A expression levels and lactate secretion in glioma cell lines HTZ-349 and U87. Glioma cell lines expressed considerable levels of LDH-A at the mRNA (Figure 1A) level. Protein expression was investigated in several glioma cell lines (HTZ-349, U87, A172), a malignant melanoma cell line (MelIm) and a prostatic cancer cell line (PC3), all expressing LDH-A at much higher levels as human fibroblasts (Figure 1B). The expression of LDH-A and LDH-B did not differ under starving conditions and conditions using 5% of FCS (not shown). Levels of lactate, secreted to the supernatants of these cell lines (Figure 1C), correlate to LDH-A expression, therefore suggesting also relevant activity of LDH-A. Lactate concentration was measured at a cell number of 2*10^5^ cells/well to avoid confounding by experimental variations. Fibroblasts and brain from hippocampal resections in epileptic patients were used as control and showed considerable lower expression of LDH-A (Figure 1A) and lactate secretion (Figure 1C). 

### Regulation of glioma cell migration by lactate

**Figure 2:**
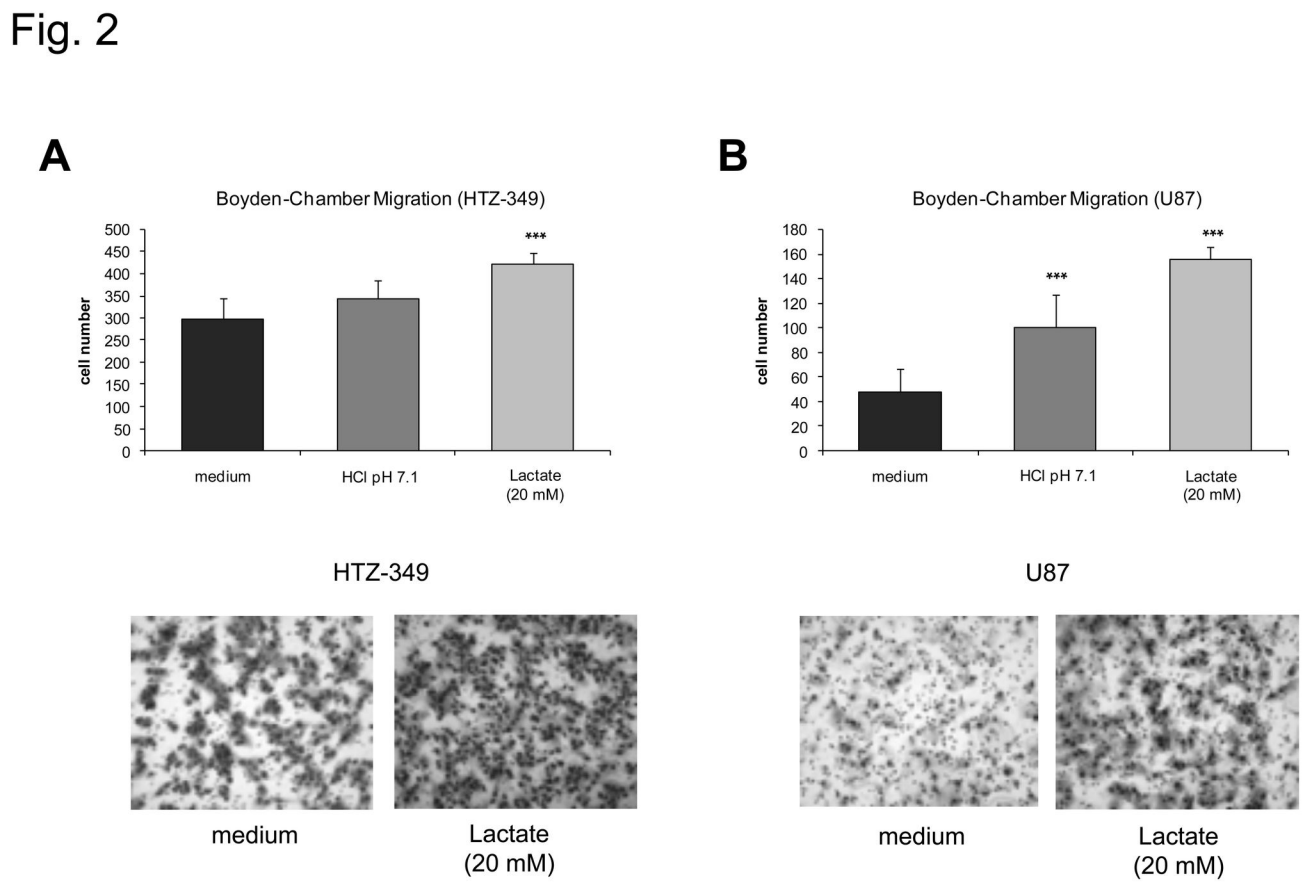
Glioma cell migration is enhanced after treatment with sodium lactate. HTZ-349 (A) and U87 (B) glioma cells were treated with 20 mM sodium lactate (pH 7.4) and HCl (pH 7.1) was used as control. 24 hours after treatment cell migration was analyzed using Boyden Chamber assays. The Y-axis indicates number of migrated cells. Treatment with lactate leads to a highly significant increase in glioma cell migration (U87 and HTZ-349, p < 0.001***).

Migration is a key feature of glioma malignancy. To evaluate if lactate is able to modulate glioma cell migration, we performed Boyden Chamber assays with U87 and HTZ-349 glioma cells. Treatment of U87 or HTZ-349 glioma cells with 20 mM sodium lactate corresponds to levels measured in brain tumor specimen^9^ and led to a highly significant increase in glioma cell migration 24 hours after treatment (Figure 2A, B). Hydrochloric acid alone failed to significantly enhance HTZ-349 glioma cell migration (Figure 2A), whereas U87 glioma cells also showed moderately increased migratory capacity after acidification by hydrochloric acid (Figure 2B). 

**Figure 3:**
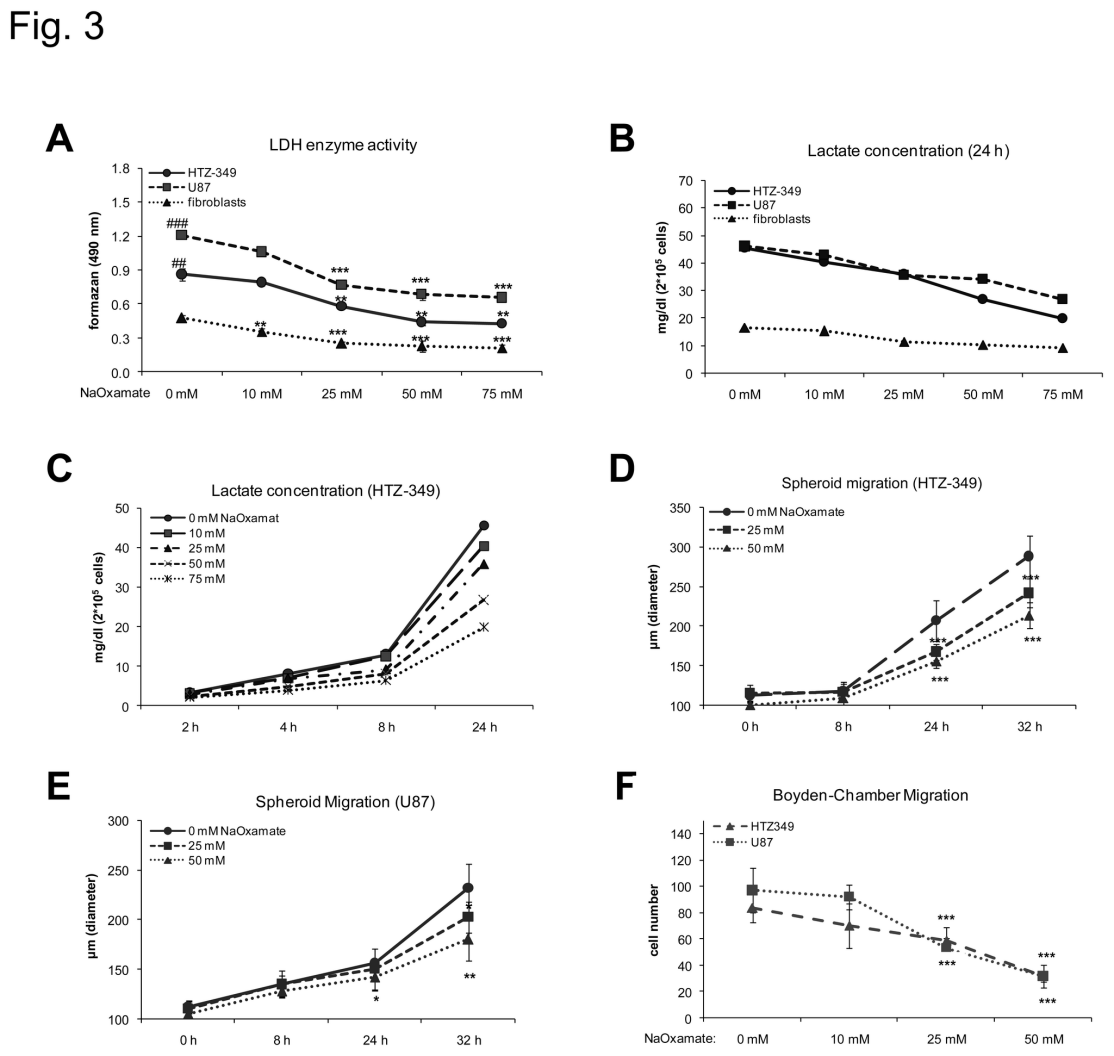
Glioma cell migration is reduced after competitive inhibition of LDH-A. Sodium oxamate was used for competitive inhibition of LDH. LDH activity was determined using the cytotox assay (Promega, Germany). Treatment with increasing doses of sodium oxamate (5 mM-75 mM) significantly reduces LDH activity 24 hours after treatment (HTZ, 349 p < 0.01** for 25mM, 50 mM and 75 mM sodium oxamate; U87, p < 0.001*** for 25mM, 50 mM and 75 mM sodium oxamate, fibroblasts, p < 0.01** for 10 mM and p < 0.001*** for 25mM, 50 mM and 75 mM sodium oxamate). HTZ 349 and U87 showed significantly higher LDH activity compared to fibroblasts (HTZ-349 p < 0.01^##^, U87 p < 0.001^###^) (A). Lactate levels were measured in cell culture supernatants 24 hours after treatment with increasing doses of sodium oxamate (10 mM-75 mM). Treatment with sodium oxamate leads to a dose- (B) and time-dependent reduction (C) of extracellular lactate levels. Treatment with 25 mM and 50 mM sodium oxamate significantly reduces HTZ-349 (D) and U87 (E) glioma cell migration starting 24 hours after treatment (HTZ, 349 p < 0.001*** at 24 h and 32 h for 25 and 50 mM sodium oxamate; U87, p < 0.05* at 24 h, p< 0.01** at 32 h for 50 mM sodium oxamate). (F) Boyden chamber assay showing similar effects as in (D) and (E) (U87, p< 0.001***; HTZ-349, p < 0.001***). The Y-axis indicates the number of migrated cells. Results were normalized to control.

Next, we tested how glioma cell migration is regulated after reduction of lactate levels. We therefore treated HTZ-349 and U87 glioma cells with sodium oxamate, a competitive inhibitor of LDH. As a structural analogue of pyruvate, sodium oxamate inhibits LDH V catalyzed conversion of pyruvate into lactate. Treatment with 25 mM sodium oxamate and above led to a significant 30-40% inhibition of LDH enzyme activity in HTZ-349 and U87 (Figure 3A). Increasing the dose of sodium oxamate to 50 mM enhanced LDH inhibition to 51% in HTZ-349 glioma cells and 57% in U87. Correspondingly, lactate levels decreased in a dose- and time-dependent way after treatment with sodium oxamate (Figure 3B, C). In spheroid migration assays treatment with sodium oxamate led to a dose-dependent highly significant decrease of HTZ-349 and U87 glioma cell migration 24 hours after treatment (Figure 3D, E) as well as in Boyden chamber assays (Figure 3F). To avoid confounding effects by impaired cell proliferation and apoptosis, pre-tests were performed to confirm that reduced cellular proliferation by oxamate is starting 24 hours after treatment in both cell lines, therefore supporting a true effect on migration in that assays that are consistently harvested before 24 hours (Figure S1A, S1B). 

### Influence of lactate on the expression of TGF-beta2

**Figure 4:**
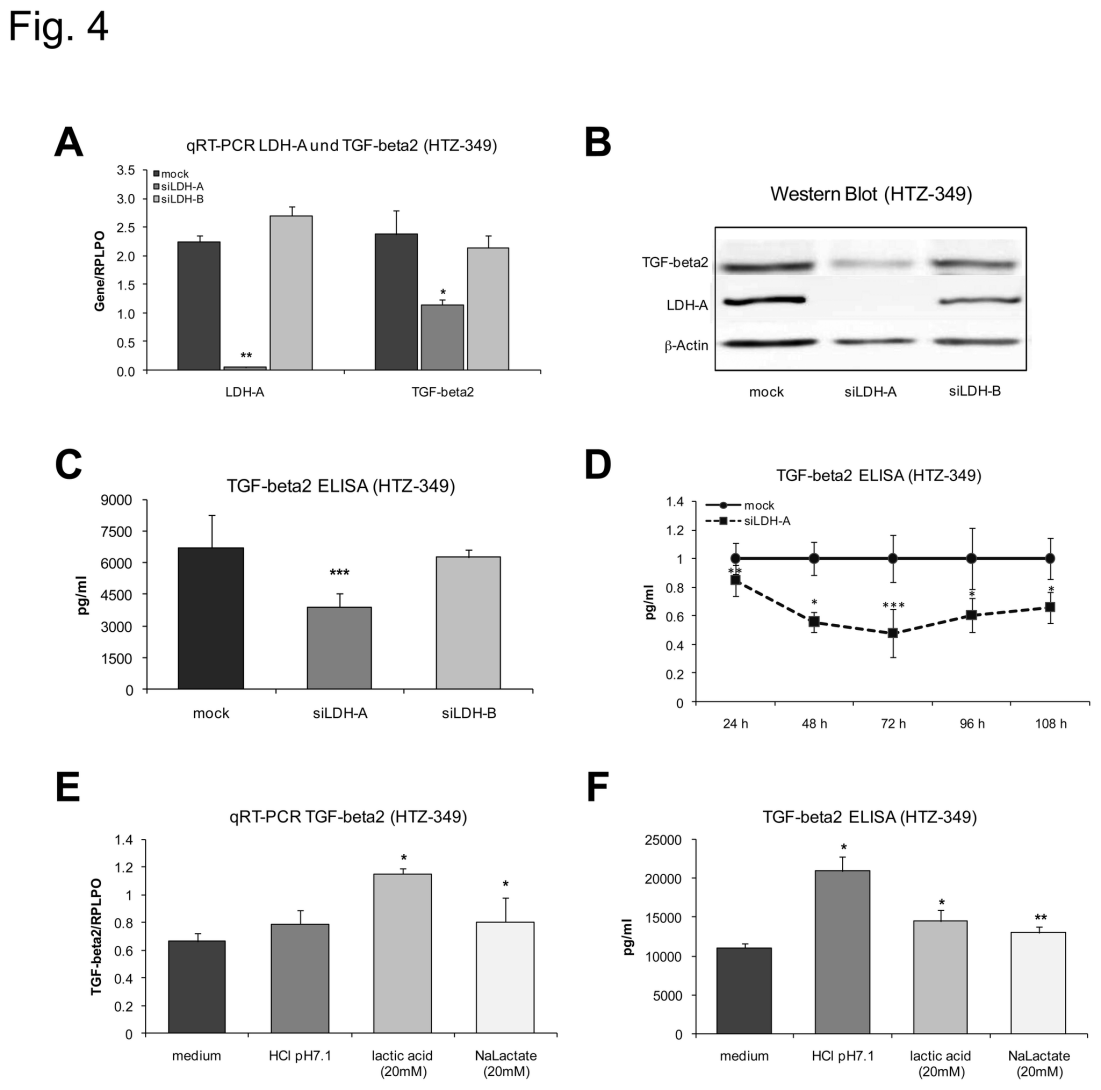
Lactate regulates TGF-beta2 protein. Specific siRNA against LDH-A was used to reduce extracellular lactate levels and to investigate consecutive changes in TGF-beta2 expression. siLDH-A reduces LDH-A (p < 0.01**) and TGF-beta2 (p< 0.05*) mRNA expression significantly 72 hours after treatment (A). In TGF-beta2 Western Blot (B) and ELISAs (C, D) siLDH-A reduces TGF-beta2 protein expression with a maximum reduction 72 hours after treatment (p< 0.001***). Results were normalized to control. Treatment with 20 mM lactic acid (pH 7.1) as well as 20 mM sodium lactate (pH 7.4) increases TGF-beta2 mRNA (E). Expression of TGF-beta2 protein 24 hours after treatment is significantly increased by lactic acid, lactate and HCl (F, lactic acid p < 0.05*; sodium lactate p < 0.01**, HCl p < 0.05*).

As TGF-beta is a known inductor of migration in glioma, we next hypothesized that lactate might induce glioma cell migration by regulation of TGF-beta2. To test this hypothesis, we regulated lactate production by transfecting cells with siLDH-A and monitored for changes of TGF-beta2 levels. Treatment of glioma cells with 0.1 µM siLDH-A specifically suppressed mRNA of LDH-A in HTZ-349 glioma cells to 2% of the initial levels (Figure 4A), leading to decreased levels of lactate and a parallel moderate increase of glucose (Figure S2A). Regulation of LDH-A RNA was followed by regulation of LDH-A but not LDH-B protein (Figure 4B). LDH-A, but not LDH-B knockdown also led to a decrease in TGF-beta2 mRNA (Figure 4A) and TGF-beta2 protein in TGF-beta2 Western blot (Figure 4B) and ELISA (Figure 4C), with a maximum regulation 72 hours after transfection (Figure 4D). Experiments in U87 yielded consensual results (not shown). These results were confirmed in RAV20 and RAV21 brain tumor initiating cells with similar results (Figure S3A).

Correspondingly, lactic acid and sodium lactate, but not hydrochloric acid significantly increased TGF-beta2 mRNA in HTZ-349 glioma cells (Figure 4E). These results were confirmed in RAV20 brain tumor initiating cells with consensual results (Figure S3B). Furthermore, TGF-beta2 protein increased modestly after treatment with sodium lactate and significantly after treatment with lactic acid and even more pronounced after treatment with hydrochloric acid after 24 hours (Figure 4F), suggesting a direct protein processing effect of acid in this context. This effect was reproduced in cells cultured with 5% FCS (Figure S2B) to exclude a starving effect. All assays were repeated in U87 with similar results (not shown).

### Regulation of THBS-1 expression by lactate

**Figure 5:**
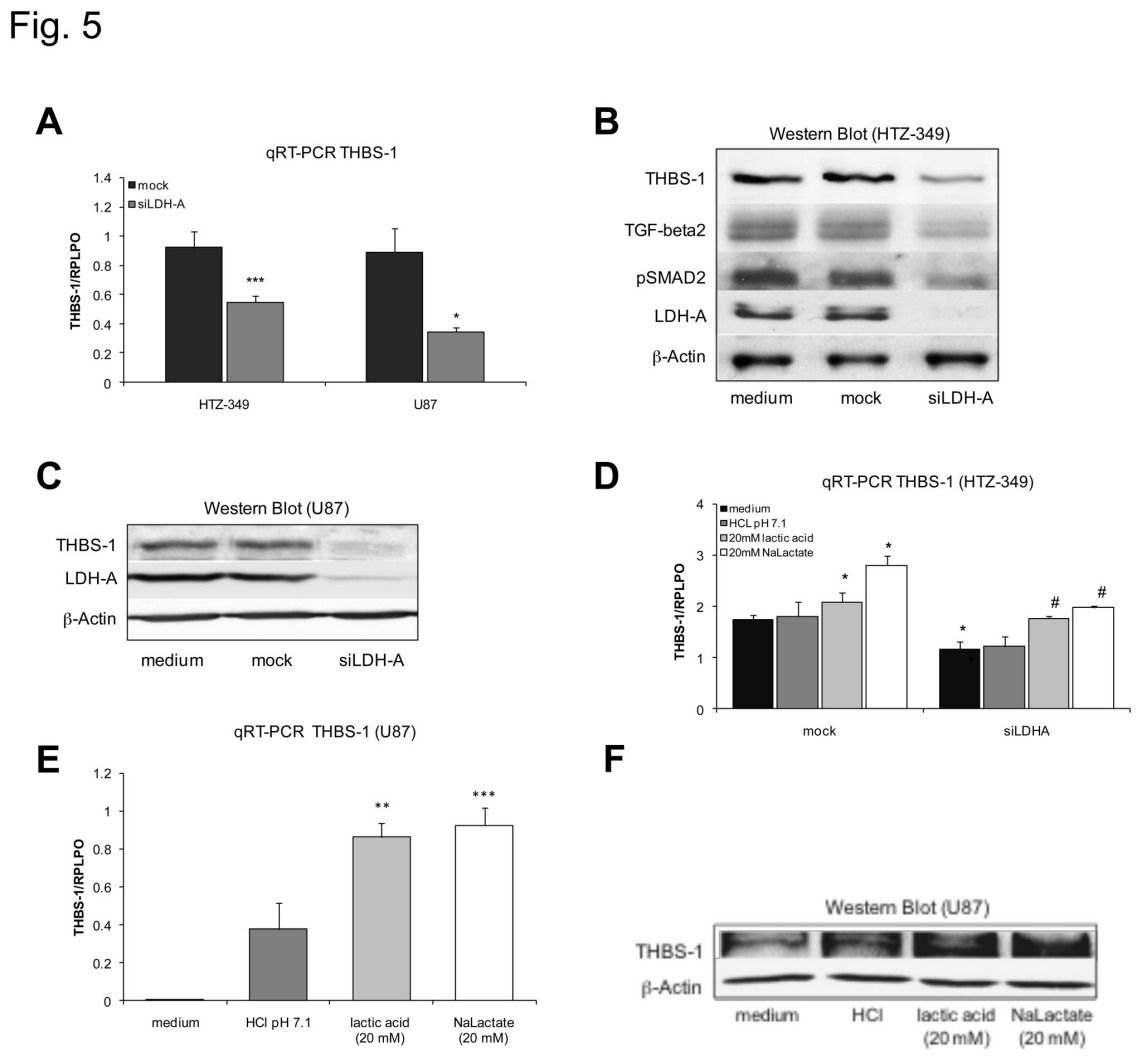
Regulation of THBS-1 expression by lactate. mRNA levels of THBS-1 were examined in qRT-PCR using specific primers for THBS-1. Treatment of glioma cells with 0.1 µM siLDH-A significantly suppresses THBS-1 at the mRNA level in HTZ-349 and U87 glioma cells (A, HTZ-349 p < 0.001***; U87 p < 0.05*). Western blot analysis was used to determine LDH-A and THBS-1 protein expression after treatment with siLDH-A. β-Actin was used as a loading control. Suppression of LDH-A also leads to a reduction of THBS-1 protein in HTZ-349 (B) and U87 (C) glioma cells. Further, TGF-beta2 expression and pSmad2 are markedly suppressed after siLDH-A treatment in HTZ-349 glioma cells (B). Treatment with 20 mM sodium lactate (pH 7.4) or 20 mM lactic acid (pH 7.1) significantly induces THBS-1 in HTZ-349 (D) and U87 (E) glioma cells at the mRNA level 24 hours after treatment (HTZ-349 p < 0.05^*^; U87 for lactic acid p < 0.01**, for sodium lactate p < 0.001***). Treatment of siLDH-A transfected HTZ-349 glioma cells with sodium lactate and lactic acid can fully rescue impaired THBS-1 expression (HTZ-349 p < 0.05^#^ for sodium lactate and lactic acid after siLDH-A transfection) (D). Western Blot analysis confirmed induction of THBS-1 protein in U87 glioma cells after treatment with lactic acid and sodium lactate (F).

As THBS-1 is a potential activator of TGF-beta2 expression in glioma lines, we next investigated the influence of LDH-A knockdown and lactate on THBS-1 expression. qRT-PCR and Western Blot analysis were performed to examine THBS-1 after siLDH-A treatment. LDH-A knockdown by siLDH-A reduced THBS-1 significantly at the mRNA (Figure 5A) and protein level (Figure 5B, C) in HTZ-349 and U87 glioma cells. Subsequently TGF-beta2 and pSmad2, a pivotal signaling molecule of the TGF-beta pathway, but not unphosphorylated Smad2 (not shown), were down-regulated (Figure 5B). Results were confirmed in RAV21 brain tumor initiating cells with consensual results (Figure S3C).

In accordance to these results treatment with 20 mM sodium lactate or 20 mM lactic acid significantly induced THBS-1 in U87 and HTZ-349 glioma cells at the mRNA level. Acidification with hydrochloric acid alone had no significant effect (Figure 5D, E). At the protein level, treatment with lactic acid and lactate, but not hydrochloric acid also induced THBS-1, indicating that the lactate anion and not acidification regulates THBS-1 expression (Figure 5F). Similar results were obtained in RAV20 brain tumor initiating cells, however, HCl yielded a significant increase of THBS-1 in these cells (Figure S3D). Treatment of siLDH-A transfected HTZ-349 glioma cells with sodium lactate and lactic acid almost fully rescued impaired THBS-1 expression confirming that lactate levels induce THBS-1 expression independent of LDH-A (Figure 5D). 

### Interaction of THBS-1 and TGF-beta2

**Figure 6:**
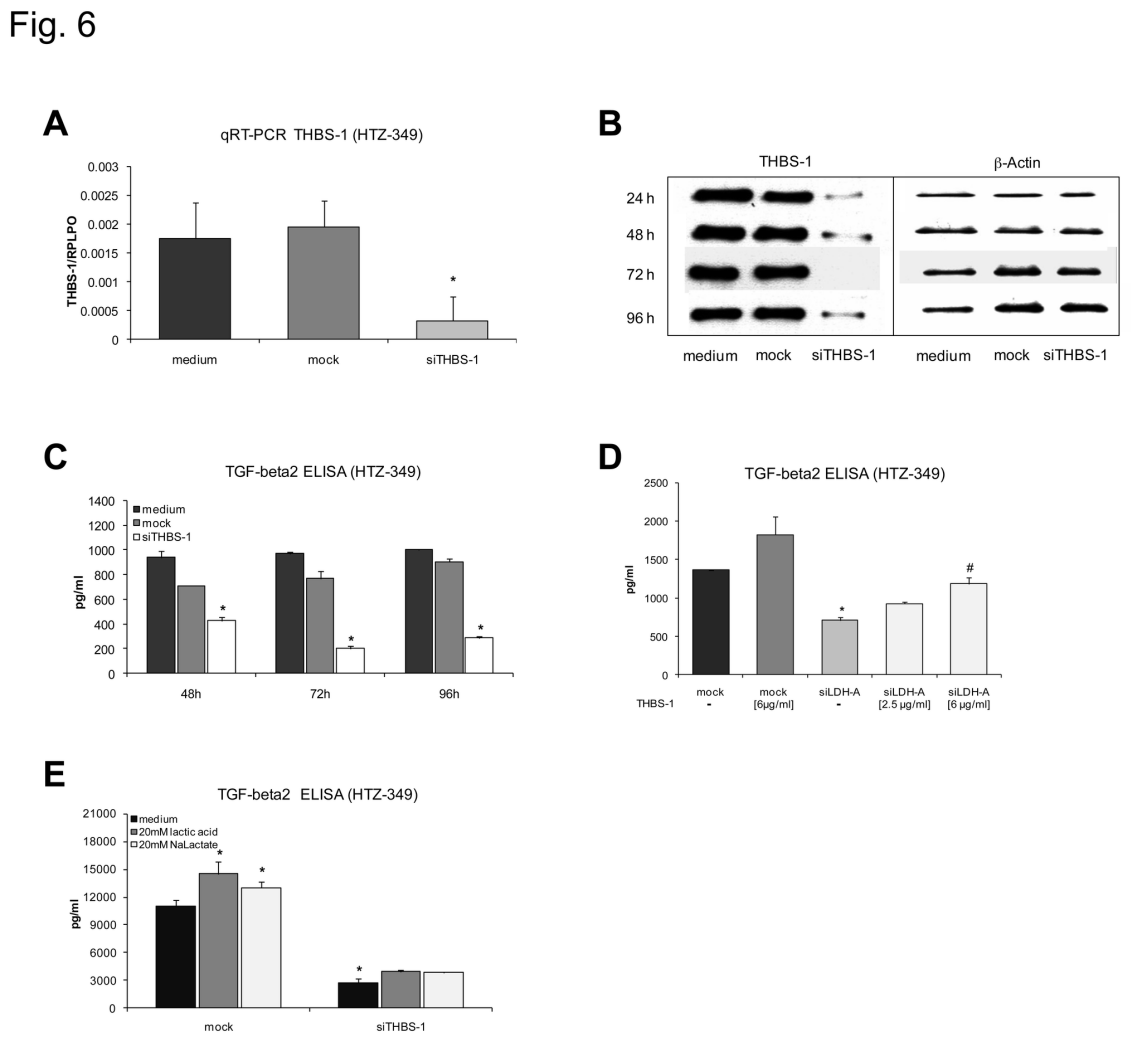
Knockdown of THBS-1 down-regulates TGF-beta2 at the protein level. 0.1 µM of siTHBS-1 significantly inhibit THBS-1 at the mRNA level as assessed in qRT-PCR (A, p< 0.05*). Western Blot analysis showed markedly reduced THBS-1 protein levels over 96 hours after treatment with siTHBS-1, with a maximum reduction 72 hours after treatment (B). In TGF-beta2 ELISAs siTHBS-1 causes significant down-regulation of TGF-beta2 protein in HTZ-349 glioma cells with a maximum reduction at 72 hours (C, p < 0.05*). Decreased levels of TGF-beta2 protein after LDH-A knockdown (D, mock/siLDH-A p < 0.05*) can be rescued by addition of increasing doses of synthetic THBS-1 protein (siLDH-A/siLDH-A+6 µg/ml THBS-1, p < 0.05^#^). Lactic acid and sodium lactate fail to significantly induce TGF-beta2 expression after transfection with siTHBS-1 (E, significance is indicated as compared to mock treated cells with standard medium, p < 0.05*).

After knockdown of LDH-A by specific siLDH-A, THBS-1 was down-regulated at the protein level (Figure 5B). In addition, TGF-beta2 as a known THBS-1 activated protein and phosphorylation of the TGF-beta signaling pathway molecule Smad2 were reduced (Figure 5B), suggesting a possible cascade effect initiated by lactate-induced regulation of THBS-1. To further elucidate the interaction between THBS-1 and TGF-beta2, we designed a specific siRNA against THBS-1, which was able to inhibit THBS-1 at the mRNA (Figure 6A) and protein level (Figure 6B) for up to 96 hours, with a maximum reduction of 95% 72 hours after transfection. Accordingly, THBS-1 knockdown led to a statistically significant 75% reduction of TGF-beta2 protein 72 hours after treatment (Figure 6C) that begins at 48 hours and holds for at least 96 hours. Notably, decreased levels of TGF-beta2 protein after LDH-A knockdown at the time of maximum regulation could be significantly rescued by addition of increasing doses of synthetic THBS-1 protein (Figure 6D), indicating a relevant activating effect of THBS-1. In addition, treatment of siTHBS-1 transfected cells with lactic acid and sodium lactate failed to relevantly activate TGF-beta2, substantiating the role of THBS-1 as a major link between lactate and TGF-beta2 (Figure 6E). All assays were reproduced in U87 with similar results (not shown). 

### Modulation of glioma cell migration by TGF-beta2 and THBS-1

**Figure 7:**
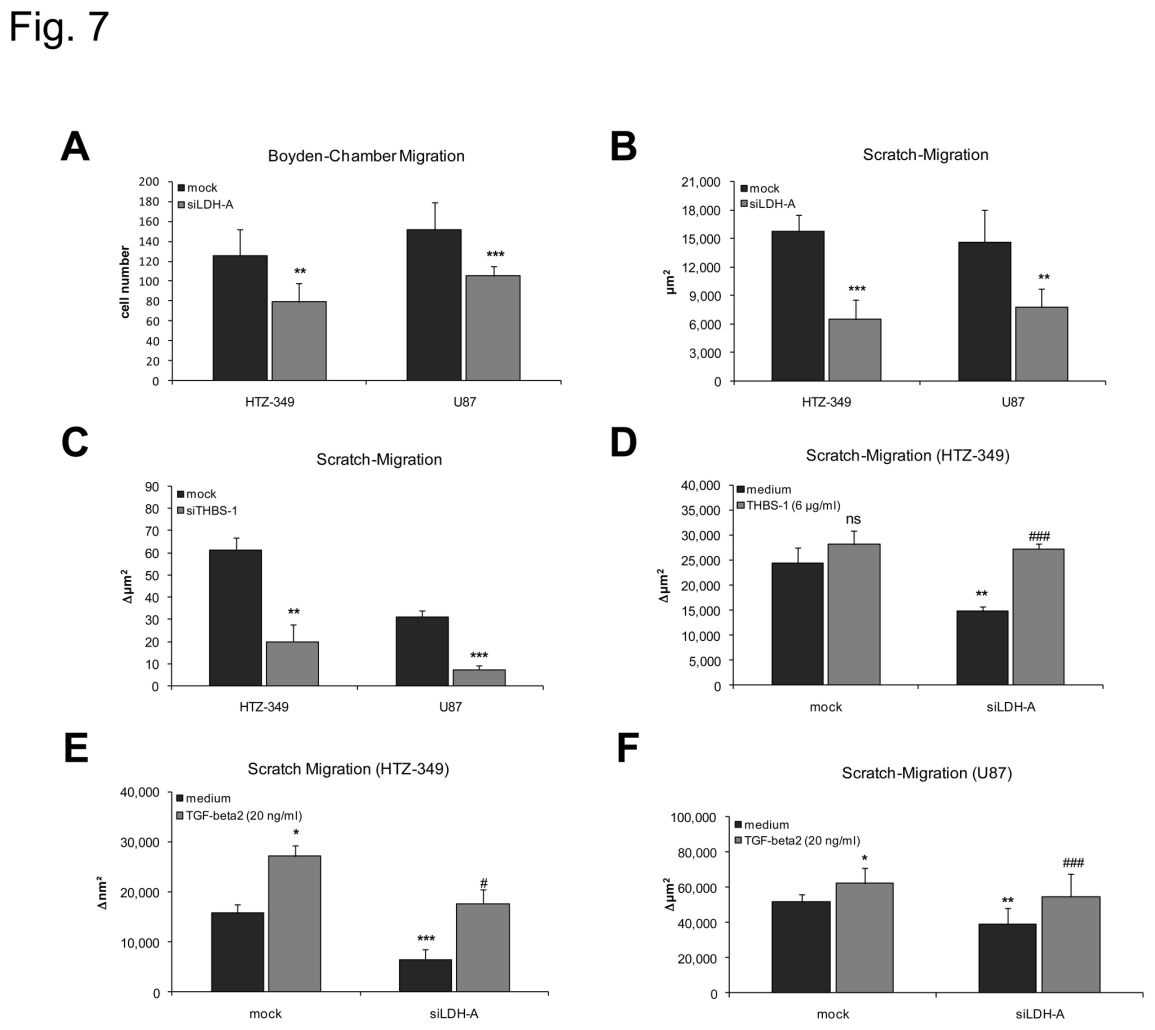
Glioma cell migration is mediated by THBS-1 and TGF-beta2. Boyden Chamber assays of HTZ-349 and U87 glioma cells 24 hours after treatment with 0.1 µM siLDH-A show a significant inhibition of migration (A, U87 p < 0.001***; HTZ-349 p < 0.01**). The Y-axis indicates the number of migrated cells. Scratch Migration assays verified these results (B-F). Here, the Y-axis indicates the area of an artificial gap in the confluent cell monolayer. Inhibition of LDH-A by siRNA yields similar results (B) as in the Boyden chamber assay. THBS-1 knockdown also diminishes HTZ-349 and U87 migration (C, HTZ-349 p < 0.01**; U87 p < 0.001***). Addition of 6 µg/ml recombinant THBS-1 (D) and 20 ng/ml TGF-beta2 (E, F) can fully rescue impaired migration after LDH-A knockdown (HTZ-349, p < 0.001*** for decrease of migration after siLDH-A, p < 0.05*^,#^ for induction of migration after treatment with TGF-beta2; U87, p < 0.01** for decrease of migration after siLDH-A, p < 0.05* for induction of migration after treatment with TGF-beta2 in mock- and p < 0.001^###^ for induction of migration in siLDHA-transfected TGF-beta2 treated cells).

To investigate the functional relevance of THBS-1 and TGF-beta2 on glioma cell migration we performed *in vitro* migration assays after differential treatment of glioma cells. First, we examined the migratory capacity of siLDH-A-transfected HTZ-349 and U87 glioma cells in Boyden (Figure 7A) and Scratch Migration Assays (Figure 7B). Both cell lines showed significantly decreased migration after knockdown of LDH-A (and therefore decreased levels of lactate) in comparison to controls. Proliferation was not significantly changed after siLDH-A treatment during the time periods used for migration assays (Figure S1C, S1D). To verify the initiating capacity of THBS-1, we examined HTZ-349 and U87 glioma cell migration after knockdown of THBS-1 (Figure 7C). Again, both cell lines exhibited significantly reduced migration after siTHBS-1 treatment in Scratch Migration Assays. Proliferation was not markedly impaired after siTHBS-1 treatment (Figure S1E, S1F). Finally, we investigated how addition of synthetic THBS-1 and recombinant TGF-beta2 mediates glioma cell migration. Addition of 6 µg/ml THBS-1 completely rescued impaired migration after LDH-A knockdown (Figure 7D). Similar results were obtained in U87 (not shown). Accordingly, addition of 20 ng/ml TGF-beta2 also fully restored reduced glioma cell migration after LDH-A knockdown in HTZ-349 (Figure 7E) and U87 (Figure 7F) glioma cells, indicating a cascade starting from lactate that is relevant for the migratory capacity of glioma cells. 

## Discussion

Glioma cell migration is a characteristic feature of glioma malignancy. It depends on several mechanisms. One of the possible mechanisms, promotion of glioma cell migration by lactic acid, has been described earlier [16]. However, other underlying mechanisms are still poorly understood. In this work, we show for the first time that in addition to TGF-beta itself, the TGF-beta activating protein THBS-1 is induced by lactic acid and sodium lactate leading to increased levels of TGF-beta2 and enhanced glioma cell migration *in vitro*. 

Regulation and activation of TGF-beta underlie complex mechanisms. TGF-beta is synthesized as pro-TGF-beta consisting of the mature 24-kDa TGF-beta dimer and the latency-associated protein (pro-region, LAP). In order to resolve the non-covalent binding between TGF-beta and LAP and to activate TGF-beta, activating molecules such as plasmin, thrombospondin or integrins are needed. Also reactive oxygen species and acidification has been shown to dissolve the non-covalent binding between TGF-beta and LAP [31]. 

Seen in this context, our finding that addition of hydrochloric acid leads to increased protein, but not mRNA expression of TGF-beta2 is plausible. Also increased migratory capacity after acidification with hydrochloric acid is in line with increased activation of TGF-beta2, a known pro-migratory molecule [12,13]. TGF-beta activating effects of lactic acid can also be explained by intracellular acidification, since the dissociation constant (pKa) for lactic acid is 3.87 (298,15 K) and lactic acid will therefore rapidly dissociate into lactate and protons under experimental conditions. 

However, for the first time, we also demonstrated an activating effect of sodium lactate on TGF-beta2 mRNA and protein as well as on glioma migration. To explain this finding we examined the TGF-beta activating molecule THBS-1, which we found to be decreased after siLDH-A treatment. Interestingly, we could find an increase of THBS-1 mRNA and protein expression after treatment with sodium lactate and lactic acid, but not after acidification with hydrochloric acid, therefore providing a possible explanation of the observed effects of sodium lactate on TGF-beta2 via its activator protein. 

It has been shown earlier that lactate modulates the binding capacity of AP1 on its consensus sequence by changing the redox state of the cell. The mentioned redox state change modulates the expression of target genes of AP1, for example *ETS1* and *THBS-1* [32,33]. ETS1 is known to induce transcription together with AP1. AP1 itself binds to the promoter of *THBS-1* [34], and binding motifs for ETS1 suggest a functional role of ETS1 at the *THBS-1* promoter (affinity prediction of more than 0.95 by Genomatix search). We therefore hypothesize that induction of THBS-1 by lactate is mediated by altered THBS-1 transcription, a mechanism that that is currently further explored. 

Induction of THBS-1 with consecutively increased activation of TGF-beta2 is therefore one possible mechanism of enhanced TGF-beta2 protein expression at increased levels of sodium lactate. To explain the observed effects on TGF-beta2 mRNA level known autocrine effects of TGF-beta have to be considered. TGF-beta2 secreting cells mostly also posses TGF-beta receptors and therefore increased levels of active extracellular TGF-beta2 enhance transcription of pro-TGF-beta [35,36]. In addition, the above mentioned link between lactate and AP-1 might also explain effects on TGF-beta mRNA, since AP-1 also binds to the promoter of TGF-beta [37]. 

Since a fundamental hypothesis of this work was that increased levels of THBS-1 lead to increased activation of TGF-beta2, a mechanism that has been evaluated mostly for TGF-beta1, we had to confirm activation of TGF-beta2 by THBS-1 in our cellular model. We were able to show that knockdown of THBS-1 significantly reduces TGF-beta2 on the protein level. On the other hand, addition of synthetic THBS-1 increases TGF-beta2 protein. Activation of TGF-beta1 by THBS-1 is currently understood as a two-step mechanism. First, the WSXW sequence of THBS-1 allows orientation of latent TGF-beta, so that the KRFK sequence of THBS-1 is associated to the corresponding LSKL sequence of the latency associated peptide, allowing proteolytic cleavage from mature TGF-beta [24,38,39]. Recent publications indicate that this mechanism could also apply for TGF-beta2. Zhang et al. observed that activation of TGF-beta2 in endothelial cells by hypoxia is dependent on THBS-1 [40]. Additionally, Ribeiro et al. were able to show that the LSKL sequence of pro-TGF-beta2 is conserved in all TGF-beta isoforms and that THBS-1 activates TGF-beta2 in Chinese hamster ovarian cells [25]. We therefore postulate that lactate mediated induction of THBS-1 leads to increased proteolytic cleavage of TGF-beta2, leading to enhanced expression of TGF-beta2. 

One of the functional experimental readouts of invasion is migration of glioblastoma cells *in vitro*. Scratch, Boyden or spheroid migration assays are reliable methods providing quantitative results. According to our results, a knockdown of LDH-A by siRNA with consecutive decreases in lactate levels leads to an approximately 40% decrease in migration compared to glioma cells treated with a control siRNA. This effect can be completely rescued by addition of synthetic THBS-1 and recombinant human TGF-beta2. TGF-beta2 is a known activator of glioma cell migration and invasion [12,13]. THBS-1 has also shown to be an activator of glioma cell migration through TGF-beta2 dependent and independent ways. THBS-1 has proven to reduce degradation of uPA by preventing association of uPA, uPAR and PAI-1, resulting in increased migration of tumor cells [41,42]. Also, activation of MMP-2 by THBS-1 in smooth muscle cells is currently under discussion [43]. We therefore conclude that lactate, by induction of THBS-1, modulates migration of glioma cells *in vitro* through TGF-beta2 dependent and independent ways. 

It is important to state, that the observed effects of lactate on THBS-1 and TGF-beta2 are obtained in a highly artificial cellular model, using established glioma cell lines. In order to verify results in a model closer to the *in vivo* conditions, we repeated our core results in brain tumor initiating cells. Besides, control for confounding effects such as altered proliferation, apoptosis or energy supply after regulation of lactate has to be discussed, although we did not observe significant changes in cellular proliferation and ATP production after siLDH-A transfection in the time periods used for migration assays (not shown). Also, the level of lactate transporters, such as MCT-1 and MCT-4, could be affected by lactate supplementation to cell culture and should be examined in more detail in further studies [44]. Additionally, the observed effects of lactate on TGF-beta2 are moderate, however, it is important to state that the effects of TGF-beta are context- and concentration-dependent, meaning that the demonstrated level of regulation can well induce meaningful functional effects [45]. We did not examine TGF-beta1 in detail, since TGF-beta2 is known to be the more relevant cytokine in glioma biology [46].

Summarized, our results suggest additive effects of lactate and lactic acid on TGF-beta2 and glioma migration: a processing effect of protonated lactate on TGF-beta2 protein via acidification and a cascade effect where lactate induces TGF-beta2 activation via induction of THBS-1. Processing of TGF-beta by THBS-1 has been described earlier [24]. However, an effect of aerobic glycolysis on THBS-1 / TGF-beta2 has not been shown yet. TGF-beta2 is one of the prominent modulators of glioblastoma pathogenesis. A link to glycolysis therefore provides another important mechanism broadening the pathophysiological potential of this pivotal protein.

## Supporting Information

Figure S1 **Control for contributory effects.** Proliferation of HTZ-349 and U87 glioma cells was assessed after treatment with sodium oxamate, siLDH-A and siTHBS-1. Trypan Blue was used to control for cell viability. Treatment with sodium oxamate (A, B) and LDH-A (C, D) significantly (p < 0.05* or p < 0.01**) reduces cell proliferation starting 24 hours after treatment in both cell lines. Treatment with siTHBS-1 did not show a significant effect on glioma cell proliferation (E, F).
(TIFF)

Figure S2 **Control for contributory effects.** 24 hours after transfection of HTZ-349 with siLDH-A, lactate levels in the cell culture supernatant decrease significantly (A; p = 0.05*), accompanied by an as well significant increase of extracellular glucose (p = 0.05*) as assessed by mass spectrometry according to [16]. In addition, we investigated the effect of fetal calf serum (FCS) on the regulation of TGF-beta after transfection with siLDH-A to exclude a starving effect (B). As in the assay using 0% FCS (Figure 4F), a slight induction of TGF-beta2 protein after treatment with lactic acid and NaLactate could be detected under culturing conditions with 5% FCS.
(TIFF)

Figure S3 **Brain tumor initiating cells.** Results were repeated in brain tumor initiating cells. LDH-A knockdown with 0.1 µM siLDH-A leads to a decrease of TGF-beta2 mRNA in RAV20 and RAV21 brain tumor initiating cells (A, p < 0.05*). Treatment of RAV-20 with lactic acid (pH 6.4) and sodium lactate (pH 7.4), but not HCl, leads to an induction of TGF-beta2 mRNA in qRT-PCR 24 hours after treatment (B, lactate p < 0.05*; lactic acid p < 0.01**). Down-regulation of LDH-A and THBS-1 by siRNA leads to significantly reduced mRNA levels (C, siLDH-A p < 0.01**; siTHBS-1 p < 0.01**). Finally, treatment of RAV20 with HCl (p < 0.01**), sodium lactate (pH 7.4; p < 0.001***) and lactic acid (pH 6.4; p < 0.001***) significantly increases THBS-1 mRNA level in qRT-PCR (D).
(TIFF)

## References

[1] Warburg O (1956) On respiratory impairment in cancer cells. Science 124: 269-270. PubMed: 13351639. 13351639

[2] Warburg O (1956) On the origin of cancer cells. Science 123: 309-314. doi:10.1126/science.123.3191.309. PubMed: 13298683. 13298683

[3] Derda DF, Miles MF, Schweppe JS, Jungmann RA (1980) Cyclic AMP regulation of lactate dehydrogenase. Isoproterenol N 6,O2 '-dibutyryl cyclic AMP increase the levels of lactate dehydrogenase-5 isozyme and its messenger RNA in rat C6 glioma cells. J Biol Chem 255: 11112-11121 6160145

[4] Hawkins RA, Choi Y, Huang SC, Messa C, Hoh CK et al. (1992) Quantitating tumor glucose metabolism with FDG and PET. J Nucl Med 33: 339-344. PubMed: 1740699. 1740699

[5] Fulham MJ, Bizzi A, Dietz MJ, Shih HH, Raman R et al. (1992) Mapping of brain tumor metabolites with proton MR spectroscopic imaging: clinical relevance. Radiology 185: 675-686. PubMed: 1438744. 1438744 10.1148/radiology.185.3.1438744

[6] Griffiths JR (1991) Are cancer cells acidic? Br J Cancer 64: 425-427. doi:10.1038/bjc.1991.326. PubMed: 1911181. 1911181 PMC1977628

[7] Stubbs M, Rodrigues L, Howe FA, Wang J, Jeong KS et al. (1994) Metabolic consequences of a reversed pH gradient in rat tumors. Cancer Res 54: 4011-4016. PubMed: 8033132. 8033132

[8] Rofstad EK, Mathiesen B, Kindem K, Galappathi K (2006) Acidic extracellular pH promotes experimental metastasis of human melanoma cells in athymic nude mice. Cancer Res 66: 6699-6707. doi:10.1158/0008-5472.CAN-06-0983. PubMed: 16818644. 16818644

[9] Walenta S, Mueller-Klieser WF (2004) Lactate: mirror and motor of tumor malignancy. Semin Radiat Oncol 14: 267-274. doi:10.1016/j.semradonc.2004.04.004. PubMed: 15254870. 15254870

[10] Wick W, Naumann U, Weller M (2006) Transforming growth factor-beta: a molecular target for the future therapy of glioblastoma. Curr Pharm Des 12: 341-349. doi:10.2174/138161206775201901. PubMed: 16454748. 16454748

[11] Kjellman C, Olofsson SP, Hansson O, Von Schantz T, Lindvall M et al. (2000) Expression of TGF-beta isoforms, TGF-beta receptors, and SMAD molecules at different stages of human glioma. Int J Cancer 89: 251-258. doi:10.1002/1097-0215(20000520)89:3. PubMed: 10861501. 10861501

[12] Friese MA, Wischhusen J, Wick W, Weiler M, Eisele G et al. (2004) RNA interference targeting transforming growth factor-beta enhances NKG2D-mediated antiglioma immune response, inhibits glioma cell migration and invasiveness, and abrogates tumorigenicity in vivo. Cancer Res 64: 7596-7603. doi:10.1158/0008-5472.CAN-04-1627. PubMed: 15492287. 15492287

[13] Wick W, Grimmel C, Wild-Bode C, Platten M, Arpin M et al. (2001) Ezrin-dependent promotion of glioma cell clonogenicity, motility, and invasion mediated by BCL-2 and transforming growth factor-beta2. J Neurosci 21: 3360-3368. PubMed: 11331365. 11331365 10.1523/JNEUROSCI.21-10-03360.2001 PMC6762489

[14] Stiles JD, Ostrow PT, Balos LL, Greenberg SJ, Plunkett R et al. (1997) Correlation of endothelin-1 and transforming growth factor beta 1 with malignancy and vascularity in human gliomas. J Neuropathol Exp Neurol 56: 435-439. doi:10.1097/00005072-199704000-00012. PubMed: 9100674. 9100674

[15] Arslan F, Bosserhoff AK, Nickl-Jockschat T, Doerfelt A, Bogdahn U et al. (2007) The role of versican isoforms V0/V1 in glioma migration mediated by transforming growth factor-beta2. Br J Cancer 96: 1560-1568. doi:10.1038/sj.bjc.6603766. PubMed: 17453002. 17453002 PMC2359935

[16] Baumann F, Leukel P, Doerfelt A, Beier CP, Dettmer K et al. (2009) Lactate promotes glioma migration by TGF-beta2-dependent regulation of matrix metalloproteinase-2. Neuro Oncol 11: 368-380. doi:10.1215/15228517-2008-106. PubMed: 19033423. 19033423 PMC2743217

[17] Roberts DD (1996) Regulation of tumor growth and metastasis by thrombospondin-1. FASEB J 10: 1183-1191. PubMed: 8751720. 8751720

[18] Tuszynski GP, Nicosia RF (1996) The role of thrombospondin-1 in tumor progression and angiogenesis. Bioessays 18: 71-76. doi:10.1002/bies.950180113. PubMed: 8593167. 8593167

[19] Bornstein P (1995) Diversity of function is inherent in matricellular proteins: an appraisal of thrombospondin 1. J Cell Biol 130: 503-506. doi:10.1083/jcb.130.3.503. PubMed: 7542656. 7542656 PMC2120533

[20] Adams JC (2001) Thrombospondins: multifunctional regulators of cell interactions. Annu Rev Cell Dev Biol 17: 25-51. doi:10.1146/annurev.cellbio.17.1.25. PubMed: 11687483. 11687483

[21] Adams JC, Lawler J (2004) The thrombospondins. Int J Biochem Cell Biol 36: 961-968. doi:10.1016/j.biocel.2004.01.004. PubMed: 15094109. 15094109 PMC2885884

[22] Kawataki T, Naganuma H, Sasaki A, Yoshikawa H, Tasaka K et al. (2000) Correlation of thrombospondin-1 and transforming growth factor-beta expression with malignancy of glioma. Neuropathology 20: 161-169. doi:10.1046/j.1440-1789.2000.00327.x. PubMed: 11132930. 11132930

[23] Amagasaki K, Sasaki A, Kato G, Maeda S, Nukui H et al. (2001) Antisense-mediated reduction in thrombospondin-1 expression reduces cell motility in malignant glioma cells. Int J Cancer 94: 508-512. doi:10.1002/ijc.1497. PubMed: 11745436. 11745436

[24] Schultz-Cherry S, Chen H, Mosher DF, Misenheimer TM, Krutzsch HC et al. (1995) Regulation of transforming growth factor-beta activation by discrete sequences of thrombospondin 1. J Biol Chem 270: 7304-7310. doi:10.1074/jbc.270.13.7304. PubMed: 7706271. 7706271

[25] Ribeiro SM, Poczatek M, Schultz-Cherry S, Villain M, Murphy-Ullrich JE (1999) The activation sequence of thrombospondin-1 interacts with the latency-associated peptide to regulate activation of latent transforming growth factor-beta. J Biol Chem 274: 13586-13593. doi:10.1074/jbc.274.19.13586. PubMed: 10224129. 10224129

[26] Das S, Srikanth M, Kessler JA (2008) Cancer stem cells and glioma. Nat Clin Pract Neurol 4: 427-435. doi:10.1038/ncpneuro0862. PubMed: 18628751. 18628751

[27] Beier D, Hau P, Proescholdt M, Lohmeier A, Wischhusen J et al. (2007) CD133(+) and CD133(-) glioblastoma-derived cancer stem cells show differential growth characteristics and molecular profiles. Cancer Res 67: 4010-4015. doi:10.1158/0008-5472.CAN-06-4180. PubMed: 17483311. 17483311

[28] Nickl-Jockschat T, Arslan F, Doerfelt A, Bogdahn U, Bosserhoff A et al. (2007) An imbalance between Smad and MAPK pathways is responsible for TGF-beta tumor promoting effects in high-grade gliomas. Int J Oncol 30: 499-507. PubMed: 17203233. 17203233

[29] Ramm P, Bettscheider M, Beier D, Kalbitzer HR, Kremer W et al. (2011) 1H-nuclear magnetic resonance spectroscopy of glioblastoma cancer stem cells. Stem Cells Dev 20: 2189-2195. doi:10.1089/scd.2010.0567. PubMed: 21265608. 21265608

[30] Lawler J, Hynes RO (1986) The structure of human thrombospondin, an adhesive glycoprotein with multiple calcium-binding sites and homologies with several different proteins. J Cell Biol 103: 1635-1648. doi:10.1083/jcb.103.5.1635. PubMed: 2430973. 2430973 PMC2114380

[31] Annes JP, Munger JS, Rifkin DB (2003) Making sense of latent TGFbeta activation. J Cell Sci 116: 217-224. doi:10.1242/jcs.00229. PubMed: 12482908. 12482908

[32] Formby B, Stern R (2003) Lactate-sensitive response elements in genes involved in hyaluronan catabolism. Biochem Biophys Res Commun 305: 203-208. doi:10.1016/S0006-291X(03)00723-X. PubMed: 12732217. 12732217

[33] Hoffmann A, Gloe T, Pohl U (2001) Hypoxia-induced upregulation of eNOS gene expression is redox-sensitive: a comparison between hypoxia and inhibitors of cell metabolism. J Cell Physiol 188: 33-44. doi:10.1002/jcp.1092. PubMed: 11382920. 11382920

[34] Kim SA, Um SJ, Kang JH, Hong KJ (2001) Expression of thrombospondin-1 in human hepatocarcinoma cell lines and its regulation by transcription factor Jun/AP-1. Mol Cell Biochem 216: 21-29. doi:10.1023/A:1011022822077. PubMed: 11216860. 11216860

[35] Bouché M, Canipari R, Melchionna R, Willems D, Senni MI et al. (2000) TGF-beta autocrine loop regulates cell growth and myogenic differentiation in human rhabdomyosarcoma cells. FASEB J 14: 1147-1158. PubMed: 10834937. 10834937 10.1096/fasebj.14.9.1147

[36] Thériault BL, Nachtigal MW (2011) Human ovarian cancer cell morphology, motility, and proliferation are differentially influenced by autocrine TGFbeta superfamily signalling. Cancer Lett 313: 108-121. doi:10.1016/j.canlet.2011.08.033. PubMed: 21945631. 21945631

[37] Sullivan DE, Ferris M, Nguyen H, Abboud E, Brody AR (2009) TNF-alpha induces TGF-beta1 expression in lung fibroblasts at the transcriptional level via AP-1 activation. J Cell Mol Med 13: 1866-1876. doi:10.1111/j.1582-4934.2008.00647.x. PubMed: 20141610. 20141610 PMC2855747

[38] Murphy-Ullrich JE, Poczatek M (2000) Activation of latent TGF-beta by thrombospondin-1: mechanisms and physiology. Cytokine Growth Factor Rev 11: 59-69. doi:10.1016/S1359-6101(99)00029-5. PubMed: 10708953. 10708953

[39] Sasaki A, Naganuma H, Satoh E, Kawataki T, Amagasaki K et al. (2001) Participation of thrombospondin-1 in the activation of latent transforming growth factor-beta in malignant glioma cells. Neurol Med Chir (Tokyo) 41: 253-259; discussion: 11396305. 11396305 10.2176/nmc.41.253

[40] Zhang H, Akman HO, Smith EL, Zhao J, Murphy-Ullrich JE et al. (2003) Cellular response to hypoxia involves signaling via Smad proteins. Blood 101: 2253-2260. doi:10.1182/blood-2002-02-0629. PubMed: 12411310. 12411310

[41] Silverstein RL, Harpel PC, Nachman RL (1986) Tissue plasminogen activator and urokinase enhance the binding of plasminogen to thrombospondin. J Biol Chem 261: 9959-9965. PubMed: 2942536. 2942536

[42] Czekay RP, Kuemmel TA, Orlando RA, Farquhar MG (2001) Direct binding of occupied urokinase receptor (uPAR) to LDL receptor-related protein is required for endocytosis of uPAR and regulation of cell surface urokinase activity. Mol Cell Biol 12: 1467-1479. doi:10.1091/mbc.12.5.1467. PubMed: 11359936. PMC34598 11359936

[43] Lee T, Esemuede N, Sumpio BE, Gahtan V (2003) Thrombospondin-1 induces matrix metalloproteinase-2 activation in vascular smooth muscle cells. J Vasc Surg 38: 147-154. doi:10.1016/S0741-5214(02)75468-2. PubMed: 12844104. 12844104

[44] Sonveaux P, Végran F, Schroeder T, Wergin MC, Verrax J et al. (2008) Targeting lactate-fueled respiration selectively kills hypoxic tumor cells in mice. J Clin Invest 118: 3930-3942. PubMed: 19033663. 19033663 10.1172/JCI36843 PMC2582933

[45] Hau P, Jachimczak P, Schlingensiepen R, Schulmeyer F, Jauch T et al. (2007) Inhibition of TGF-beta2 with AP 12009 in recurrent malignant gliomas: from preclinical to phase I/II studies. Oligonucleotides 17: 201-212. doi:10.1089/oli.2006.0053. PubMed: 17638524. 17638524

[46] Schlingensiepen KH, Schlingensiepen R, Steinbrecher A, Hau P, Bogdahn U et al. (2006) Targeted tumor therapy with the TGF-beta 2 antisense compound AP 12009. Cytokine Growth Factor Rev 17: 129-139. doi:10.1016/j.cytogfr.2005.09.002. PubMed: 16377233. 16377233

