# Correction: Lactate-Modulated Induction of THBS-1 Activates Transforming Growth Factor (TGF)-beta2 and Migration of Glioma Cells *In Vitro*

**DOI:** 10.1371/annotation/78d10187-08e3-4a1c-9294-3da679630d00

**Published:** 2014-01-06

**Authors:** Corinna Seliger, Petra Leukel, Sylvia Moeckel, Birgit Jachnik, Claudio Lottaz, Marina Kreutz, Alexander Brawanski, Martin Proescholdt, Ulrich Bogdahn, Anja-Katrin Bosserhoff, Arabel Vollmann-Zwerenz, Peter Hau

A funding organization was incorrectly omitted from the Funding Statement. The Funding Statement should read: Part of the work was funded by an internal grant of the University Hospital Regensburg (ReForM-C), and by the Wilhelm Sander-Stiftung, Munich and Ingolstadt, Germany. The funders had no role in study design, data collection and analysis,decision to publish, or preparation of the manuscript. No additional external funding was received for this study. 

