# Supplementary figures and images for: Lactate-Modulated Induction of THBS-1 Activates Transforming Growth Factor (TGF)-beta2 and Migration of Glioma Cells In Vitro
Source: PLoS One. 2013 Nov 1;8(11):e78935. doi: 10.1371/journal.pone.0078935 (PMC3815307; doi:10.1371/journal.pone.0078935)

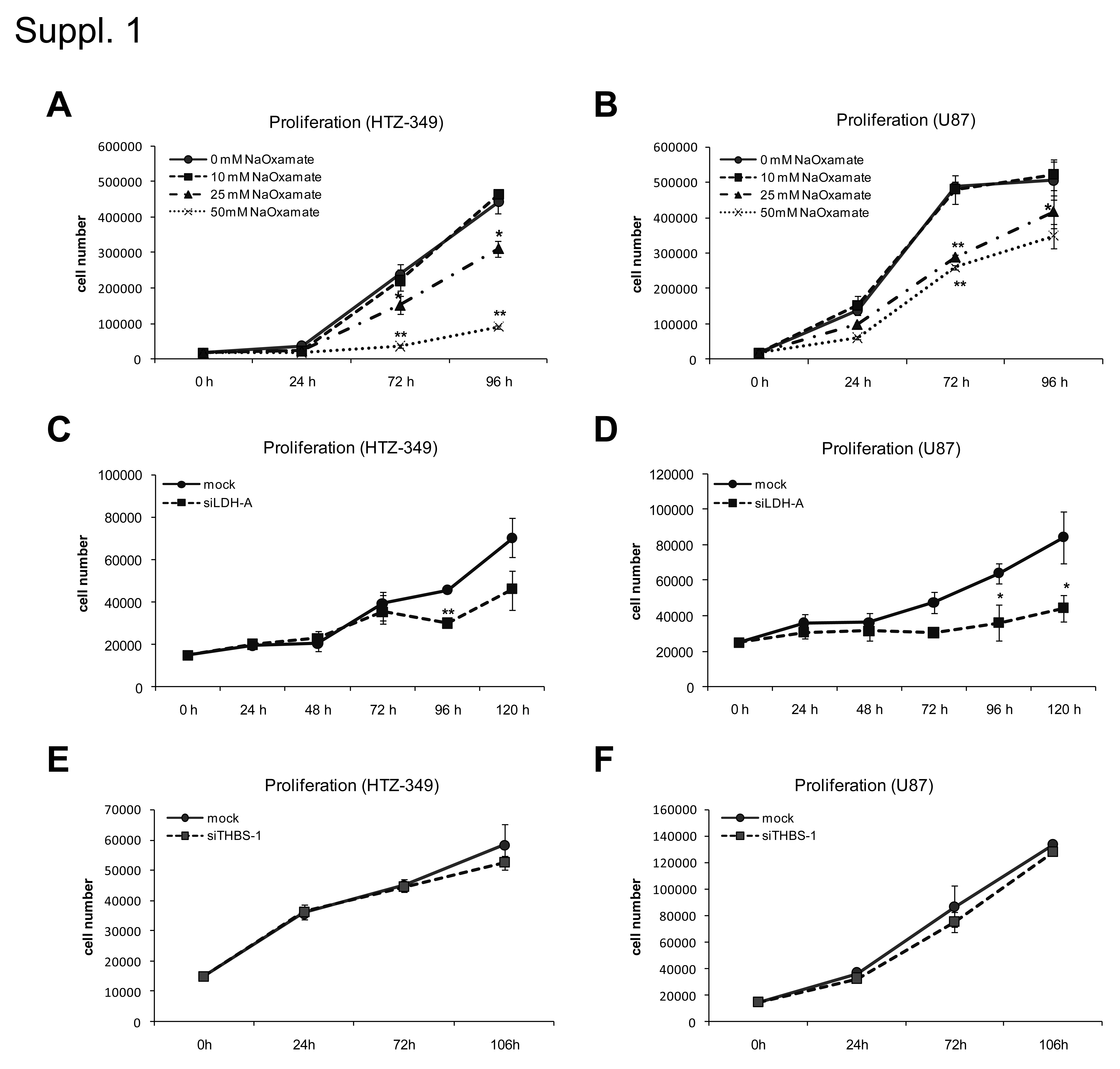

Supplement: Figure S1 — Control for contributory effects. Proliferation of HTZ-349 and U87 glioma cells was assessed after treatment with sodium oxamate, siLDH-A and siTHBS-1. Trypan Blue was used to control for cell viability. Treatment with sodium oxamate (A, B) and LDH-A (C, D) significantly (p < 0.05* or p < 0.01**) reduces cell proliferation starting 24 hours after treatment in both cell lines. Treatment with siTHBS-1 did not show a significant effect on glioma cell proliferation (E, F). (TIFF) [file pone.0078935.s001.tiff]

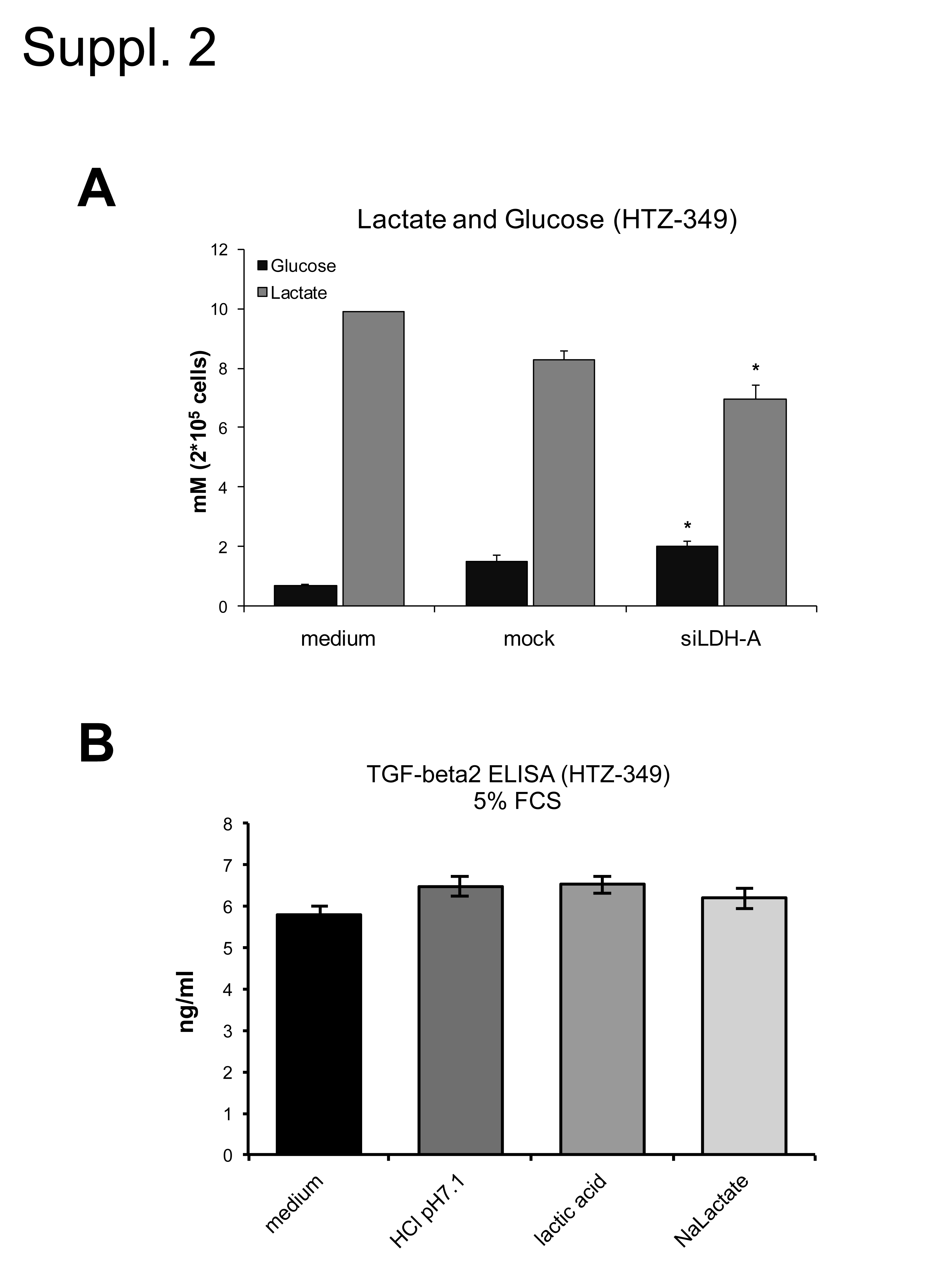

Supplement: Figure S2 — Control for contributory effects. 24 hours after transfection of HTZ-349 with siLDH-A, lactate levels in the cell culture supernatant decrease significantly (A; p = 0.05*), accompanied by an as well significant increase of extracellular glucose (p = 0.05*) as assessed by mass spectrometry according to [16]. In addition, we investigated the effect of fetal calf serum (FCS) on the regulation of TGF-beta after transfection with siLDH-A to exclude a starving effect (B). As in the assay using 0% FCS (Figure 4F), a slight induction of TGF-beta2 protein after treatment with lactic acid and NaLactate could be detected under culturing conditions with 5% FCS. (TIFF) [file pone.0078935.s002.tiff]

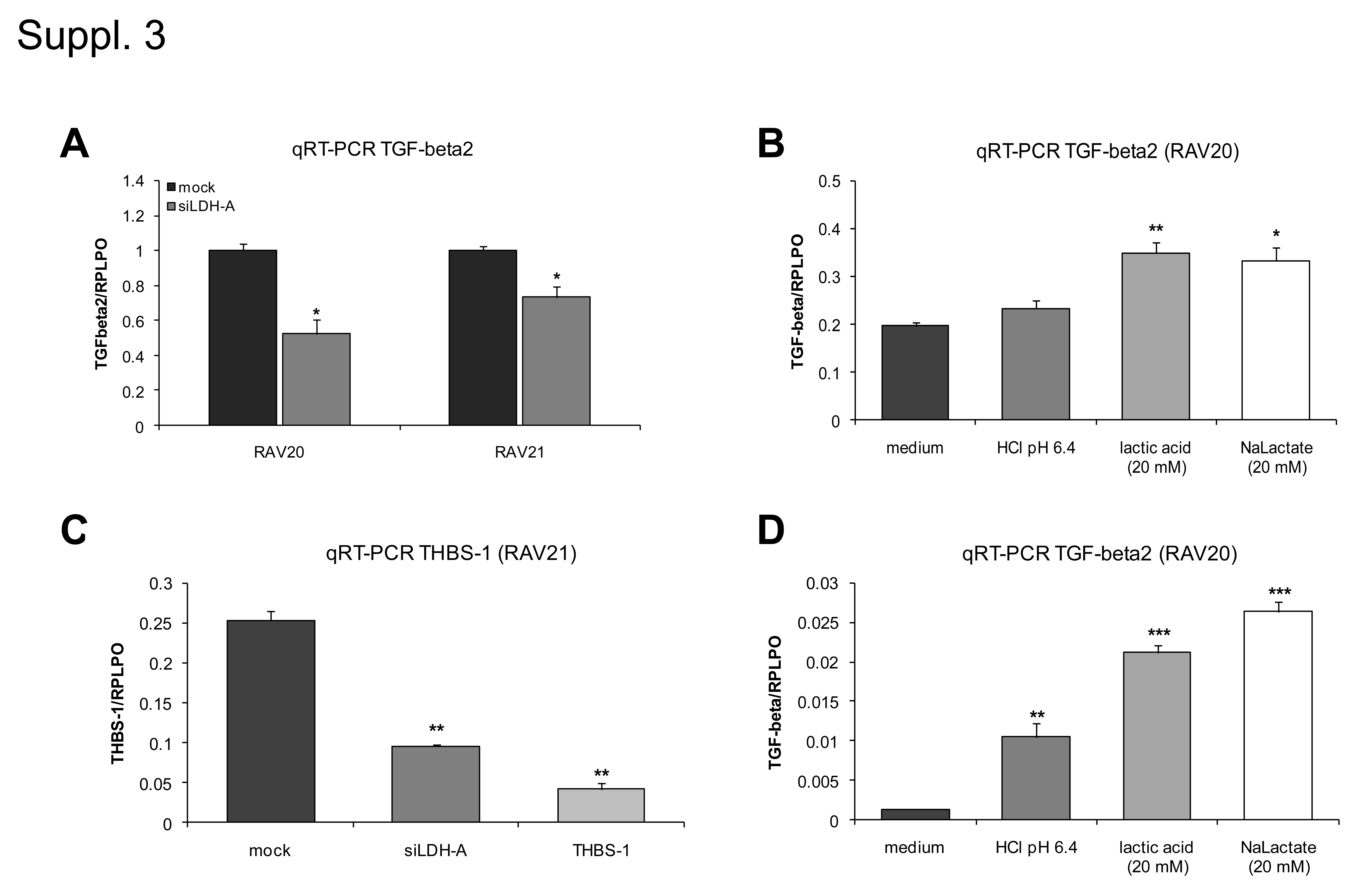

Supplement: Figure S3 — Brain tumor initiating cells. Results were repeated in brain tumor initiating cells. LDH-A knockdown with 0.1 µM siLDH-A leads to a decrease of TGF-beta2 mRNA in RAV20 and RAV21 brain tumor initiating cells (A, p < 0.05*). Treatment of RAV-20 with lactic acid (pH 6.4) and sodium lactate (pH 7.4), but not HCl, leads to an induction of TGF-beta2 mRNA in qRT-PCR 24 hours after treatment (B, lactate p < 0.05*; lactic acid p < 0.01**). Down-regulation of LDH-A and THBS-1 by siRNA leads to significantly reduced mRNA levels (C, siLDH-A p < 0.01**; siTHBS-1 p < 0.01**). Finally, treatment of RAV20 with HCl (p < 0.01**), sodium lactate (pH 7.4; p < 0.001***) and lactic acid (pH 6.4; p < 0.001***) significantly increases THBS-1 mRNA level in qRT-PCR (D). (TIFF) [file pone.0078935.s003.tiff]
